# Levels of Evidence, Quality Assessment, and Risk of Bias: Evaluating the Internal Validity of Primary Research

**DOI:** 10.3389/fvets.2022.960957

**Published:** 2022-07-12

**Authors:** Jan M. Sargeant, Marnie L. Brennan, Annette M. O'Connor

**Affiliations:** ^1^Department of Population Medicine, Ontario Veterinary College, University of Guelph, Guelph, ON, Canada; ^2^Centre for Evidence-Based Veterinary Medicine, School of Veterinary Medicine and Science, University of Nottingham, Sutton Bonington Campus, Loughborough, United Kingdom; ^3^Department of Large Animal Clinical Sciences, College of Veterinary Medicine, Michigan State University, East Lansing, MI, United States

**Keywords:** internal validity, bias, confounding, evidence-based medicine, critical appraisal, veterinary

## Abstract

Clinical decisions in human and veterinary medicine should be based on the best available evidence. The results of primary research are an important component of that evidence base. Regardless of whether assessing studies for clinical case management, developing clinical practice guidelines, or performing systematic reviews, evidence from primary research should be evaluated for internal validity i.e., whether the results are free from bias (reflect the truth). Three broad approaches to evaluating internal validity are available: evaluating the potential for bias in a body of literature based on the study designs employed (levels of evidence), evaluating whether key study design features associated with the potential for bias were employed (quality assessment), and applying a judgement as to whether design elements of a study were likely to result in biased results given the specific context of the study (risk of bias assessment). The level of evidence framework for assessing internal validity assumes that internal validity can be determined based on the study design alone, and thus makes the strongest assumptions. Risk of bias assessments involve an evaluation of the potential for bias in the context of a specific study, and thus involve the least assumptions about internal validity. Quality assessment sits somewhere between the assumptions of these two. Because risk of bias assessment involves the least assumptions, this approach should be used to assess internal validity where possible. However, risk of bias instruments are not available for all study designs, some clinical questions may be addressed using multiple study designs, and some instruments that include an evaluation of internal validity also include additional components (e.g., evaluation of comprehensiveness of reporting, assessments of feasibility or an evaluation of external validity). Therefore, it may be necessary to embed questions related to risk of bias within existing quality assessment instruments. In this article, we overview the approaches to evaluating internal validity, highlight the current complexities, and propose ideas for approaching assessments of internal validity.

## Introduction

Every day in clinical practice, veterinary professionals need to make decisions ranging from a decision as to whether (or not) to use an intervention or to apply a diagnostic test, to decisions about the overall management of complex clinical conditions. Increasingly, it is expected that clinical decisions are evidence-based. Evidence-based veterinary medicine incorporates clinician experience, client preferences, animal needs, and scientific evidence when making clinical decisions (1). In this approach, scientific evidence is obtained from relevant research. When research-based evidence does not exist, other sources of evidence, such as expert opinion may need to be used. Traditional narrative reviews provide an overview of a topic, and thus may be an attractive way of quickly acquiring knowledge for making clinical decisions. However, narrative reviews generally do not provide information on the identification and selection of the primary research being summarized (if any), the methodological quality of the studies, or the magnitude of the expected effect (2, 3).

Formal methods have been developed to systematically identify, select, and synthesize the available evidence to assist veterinary professionals in evidence-based decision-making. These include critically appraised topics (CATs) (4), systematic review and meta-analysis (SR-MA) (5–7), and clinical practice guidelines (8) (see Box 1 for a short overview of these methods). These evidence synthesis approaches have different purposes which results in different processes and endpoints, but each includes an assessment of the internal validity of the research used. Critical appraisal of an individual study also includes an evaluation of internal validity, in addition to an evaluation of feasibility and generalizability (10). The evaluation of internal validity is the focus of this article. Understanding the different ways internal validity can be assessed, and the assumptions associated with these approaches, is necessary for researchers evaluating internal validity, and for veterinary professionals to assess studies for integration of evidence into practice.

Box 1 **Overview of synthesis methods used in veterinary practice and research.**

**Systematic review, meta-analysis, and network meta-analysis:** Systematic review is a structured methodology for identifying, selecting and evaluating all relevant research to address a structured question, which may relate to descriptive characteristics such as prevalence, etiology, efficacy of interventions, or diagnostic test accuracy (5). Meta-analysis is the statistical combination of results from multiple studies. For addressing questions on intervention efficacy, meta-analysis provides an overall effect size for pairwise comparisons between two intervention groups. Network meta-analysis allows an estimation of the comparative efficacy across all available intervention options (6), which may provide more relevant information for veterinary professionals when there are multiple intervention options available. However, systematic reviews with pairwise meta-analysis or network meta-analysis require that a body of research exists that can be synthesized to address a clinical question and can also be resource and time intensive to conduct. Therefore, there are many clinical questions for which formally synthesized research summaries do not exist.

**Critically appraised topics:** Critically appraised topics (CATs) use the same principles as systematic reviews to address clinical questions but employ a more rapid approach, particularly in relation to the screening and summation of the evidence. They were designed to be employed by clinicians as a way of rapidly gathering and interpreting evidence on clinical questions relating to specific cases (4). Therefore, there is a greater risk that research addressing the question may be missed. However, in the absence of a well conducted systematic review or meta-analysis, CATs can provide a faster evaluation of research addressing a clinical question and can be undertaken by veterinary professionals who may have fewer resources and potentially less methodological or statistical expertise, particularly if they are freely available and accessible.

**Clinical practice guidelines:** Veterinary professionals often are involved in the management of complex clinical conditions, where an array of questions need to be addressed, including those related to etiology, prognosis, diagnostic test accuracy, and intervention efficacy. Clinical practice guidelines are intended to assist healthcare professionals in assessing more than one aspect of case approach, including appropriate prevention, diagnosis, treatment, or clinical management of diseases, disorders, and other health conditions (9). Although there are differences in the methods among authors and institutions, the key elements of guideline development include the establishment of a multidisciplinary working group to develop the guidelines, the involvement of appropriate stakeholders, identification of the topic area, systematic searches for research evidence, assessment of the internal validity of studies comprising the evidence base, a process for drafting recommendations, and ongoing review and updating of the guidelines as new evidence becomes available (8).

Internal validity refers to the extent to which the study results reflect the true state of nature (i.e., whether the effect size estimated in a study is free from systematic error, also called bias) (11). Although there are a large number of named biases (12), for studies that assess interventions or risk factors, the biases can be categorized into three broad types of bias: selection bias, information bias, and confounding (13). Selection bias impacts the effect size if, compared to the source population, the exposure or intervention groups differ in the distribution of factors associated with the outcome at the time the study population is selected, or if differential loss to follow up between groups occurs during the study. In case-control studies, selection bias occurs if cases or controls are selected based on criteria that are related to the exposure of interest. Information bias occurs when there are errors in measuring the exposure or intervention, or the outcome, or both. Finally, confounding is a mixing of effects that occurs when a variable (the confounder) that is independently associated with both the exposure and the outcome is not properly controlled. When confounding is not controlled, the estimate of the relationship between the exposure and the outcome will be biased.

There are several terms used to describe the approaches to assessing internal validity of primary research studies, including evidence hierarchies and levels of evidence, quality assessment, and risk of bias assessment. The use of these terms may be confusing, and it is not uncommon for some of these terms to be used interchangeably (14, 15). Also, authors may mislabel the approaches and some evaluation tools (instruments) available for assessing internal validity may include additional components, such as those related to comprehensiveness (quality) of reporting, feasibility of applying an intervention, or external validity. Finally, some instruments may use the approach as a label for the instrument [e.g., Cochrane's risk of bias tool (16), which is an instrument that employs a risk of bias approach] and other instruments may not include the approach in the instrument name [e.g., the Jadad scale (17), which employs a quality assessment approach]. In an evaluation of the comprehensiveness of reporting in animal health systematic reviews (SRs), Sargeant et al., (18)found that a range of instruments involving all three approaches had been used for assessing the internal validity of primary research studies. Although a large number of instruments are available, the approaches within each instrument used to assess internal validity can be grouped into three broad categories: based on study design, based on the presence or absence of design features, or based on a judgement about bias in the context of the study. These categories generally correspond to levels of evidence, quality assessment, and risk of bias, respectively. Therefore, our objective was to review these approaches to assessing internal validity as distinct entities and to describe the assumptions associated with each approach. Although we provide examples of specific instruments that include an evaluation of internal validity, our focus is on the approaches, rather than the tools. We discuss advances in the use of these approaches to assessing internal validity in human healthcare and propose a process for veterinary medicine for selecting the approach with the least assumptions as appropriate to the clinical question, the purpose of the assessment, and the research found that addresses the question of interest. The target audience for this article is individuals who assess internal validity of studies, individuals who develop instruments that include items related to the assessment of internal validity, and those who use evidence synthesis products created by others, such as systematic reviews or clinical practice guidelines.

### Evaluating Internal Validity by Study Design: Levels of Evidence

Levels of evidence is an approach to evaluating the internal validity of a body of evidence, based on the potential for bias which is inherent to the employed study designs that were used to address the clinical question. The concept behind levels of evidence is that there is a hierarchy of study designs, with different study designs having different potential for bias. The way evidence hierarchies are used is based on either the name of the design or the description of the design. Readers of a study look for this information, then determine the design and assign a level of evidence. No further differentiation of methodological features or judgment is conducted.

**Figure 1:**
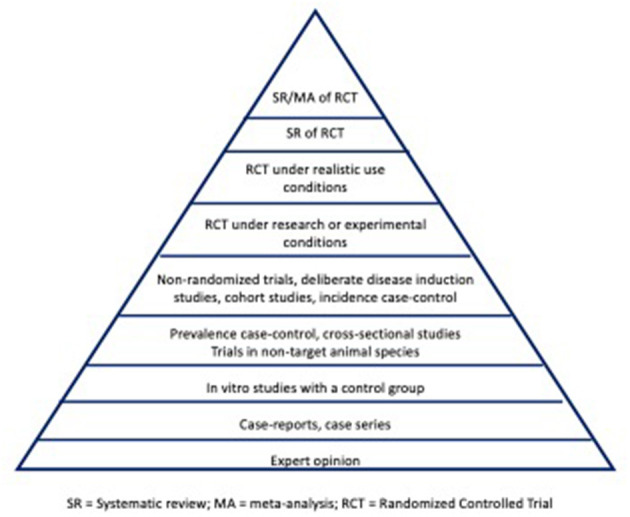
Illustration of an evidence pyramid hierarchy for addressing intervention studies in veterinary medicine. SR, systematic review; MA, meta-analysis; RCT, randomized controlled trial.

Evidence hierarchies were initially introduced in 1979 by the Canadian Task Force on the Periodic Health Examination (19), with further development into an evidence pyramid by David Sackett in 1989 (20). A pyramid shaped figure commonly is used to illustrate the hierarchy of study designs for evaluating the efficacy of an intervention under realistic-use conditions (owned animals, as opposed to experimental settings), with the potential for bias decreasing from the base to the top of the pyramid (Figure 1). Thus, study designs on the top of the pyramid represent those with inherently lower risk of bias compared to study designs lower on the hierarchy. The pyramid shape acknowledges that the quantity of research tends to decrease in the higher levels of evidence (for instance, there will be a larger volume of randomized controlled trials (RCTs) compared to SR-MA). Suggested modifications to the evidence pyramid for veterinary intervention studies include dividing RCTs into those conducted under realistic-use conditions vs. those conducted in nonrealistic-use conditions (e.g., research facility) (21), the inclusion of challenge trials (where disease outcomes are deliberately induced) below RCTs in the pyramid (21, 22), and increasing the interpretability of the concept for students by displaying the hierarchy as a staircase rather than a pyramid (23).

The concept of evaluating the potential for bias in an individual study based on the study design can be extended to an evaluation of the potential for bias in a body of literature. This approach for evaluating the internal validity of a body of literature is referred to as “levels of evidence”. The approach is applied by identifying research (or other evidence) that pertains to the clinical question, determining the study design used for each of the studies, and then assigning each study to a level of evidence based on that design. For instance, a framework for levels of evidence in veterinary clinical nutrition has been proposed by Roudebush et al. (24). In this framework, level 1 evidence corresponds to at least 1 appropriately designed RCT in the target species with natural disease development, level 2 evidence would correspond to RCTs in laboratory settings with natural disease development, level 3 evidence would be obtained from non-randomized trials, deliberate disease induction trials, analytical observational studies or case series, and level 4 evidence would correspond to expert opinion, descriptive studies, studies in other species, or pathophysiological justification. Therefore, if the clinical question involves interventions, and the evidence found to address the question consists of 2 RCTs, 3 case-control studies, and 3 case series, the evidence would be designated as “level 1 evidence” because study designs with the highest evidentiary level in the available research consisted of RCTs. If all available evidence was from expert opinion, the body of research would comprise “level 4” evidence. This evidence would represent the best available evidence to inform decision-making at the time the assessment was made, although the overall level assigned would change as higher evidentiary level information becomes available.

The levels of evidence approach may be perceived as a quick and easy approach to assessing internal validity because it requires only a knowledge of the study design employed and not the individual features of a study that may or may not be associated with the potential for bias. However, that ease of use is based on very strong assumptions: 1) that study design maps directly to bias, 2) that authors always correctly label study designs, and 3) that authors execute and report study designs appropriately. The approach also pertains to a body of evidence, implying that there are multiple comparable studies available to address the question of interest.

An important critique of levels of evidence is that the approach focuses on the study design, rather than the actual design features that were used or the context of the study. Thus, although this framework illustrates the inherent potential for bias of the different study designs, it does not provide a consideration of the methodological rigor with which any specific individual study was conducted (25). For instance, although a well-conducted cohort study may be less biased than a poorly executed RCT, this nuance is not captured by a levels of evidence approach. Additionally, levels of evidence are based on the potential for confounding and selection biases, but there is no mechanism to evaluate the potential for information bias because this is linked to the outcome and the levels of evidence approach is based on features at the study, rather than outcome, level. For instance, RCTs provide a higher level of evidence compared to observational studies because random allocation to intervention groups minimizes the potential for confounding, and case-control studies provide a lower level of evidence than cohort studies because they are more prone to selection bias. However, a RCT that used a subjectively measured outcome would be assigned a higher level of evidence than a cohort study with an objective outcome, although the observational study may have a lower risk of information bias. Finally, studies may be mislabeled in terms of their study design; there is empirical evidence that this occurs in the veterinary literature (26–28). For example, studies labeled as case series in veterinary medicine frequently include a component corresponding to a cohort study design (27); these studies may be assigned an inappropriately low level of evidence if individuals classifying these studies rely on authors terminology rather than the complete design description to determine the design employed.

An additional consideration is that for questions related to aspects of clinical care other than selection of interventions, the framework and positioning of study designs included in Figure 1 may not be appropriate. Levels of evidence schema are available for other clinical questions, such as prognosis, diagnostic test accuracy, disease screening, and etiology (29, 30).

### Evaluating Internal Validity Based on Inclusion of Study Features Associated With Bias: Quality Assessment

As the name implies, quality assessment represents an evaluation of the quality of a primary research article. However, the term “quality” is difficult to specifically define in the context of evidence-based medicine, in that it does not appear to have been used consistently in the literature. The Merriam-Webster dictionary defines quality as “how good or bad something is” or “a high level of value or excellence” (https://www.merriam-webster.com/dictionary/quality). Quality generally is understood to be a multi-dimensional concept. While clear definitions are difficult to find in the research literature, the lay literature includes numerous treaties on the dimensions of quality. One example is the eight dimensions of quality delineated by David Gavin, which include performance, features, reliability, conformance, durability, serviceability, aesthetics, and perceived quality (https://en.wikipedia.org/wiki/Eight_dimensions_of_quality).

The findings from a review (31) identified that available instruments labeled as quality assessment tools varied in clarity and often involved more than just assessing internal validity. In addition to including an assessment of internal validity, quality assessment instruments also generally contain elements related to quality of reporting or an assessment of the inclusion of study features not directly related to bias, such as whether ethical approval was sought or whether the study participants were similar to those animals in the care of the individual doing the critique (14, 31–33).

Quality assessment as an approach to evaluating internal validity involves an evaluation of the presence or absence of design features, i.e., a methodological checklist (14, 15). For example, the Jadad scale (17) involves completing a checklist of whether the study was described as randomized, whether the study was described as double blind, and whether there was a description of withdrawals and dropouts, with points assigned for each category. Therefore, the Jadad scale uses a quality assessment approach to evaluating internal validity. In terms of assumptions, the quality assessment approach also makes strong assumptions, although these are less than those used in levels of evidence assessments. Instead of mapping bias to the study design, quality assessment maps bias to a design feature i.e., if a trial was randomized, it is assumed to be “good quality” and if the trial was not randomized the assumption is that it is “poor quality”. The same process is followed for additional study aspects, such a blinding or losses to follow-up, and an overall assessment of quality is then based on how the study 'performs' against these questions.

Quality assessment also considers more than just confounding and selection bias as components of internal validity. The inclusion of blinding as a design feature of interest illustrates this. Blinding as a design feature is intended to reduce the potential for differential care as a source of confounding bias (blinding of caregivers) or may be intended to reduce the potential for information bias (blinding of outcome assessors). Conducting a quality assessment is more complicated and time-consuming than evaluating levels of evidence because the presence or absence of the specific design features needs to be identified and validated within the study report. However, the approach requires only that the person evaluating internal validity can identify whether (or not) a design feature was used. Therefore, this approach requires more technical expertise that the levels of evidence approach, but less than the risk of bias approach.

### Evaluating Internal Validity by Making Contextualized Judgements on Potential Occurrence of Bias: Risk of Bias Assessment

Risk of bias assessments have been developed specifically for evaluating the potential for elements of the design or conduct employed within a study to lead to a biased effect size (34, 35). The components of risk of bias assessments are selected based on empirical evidence of their association with estimates of effect sizes (24, 32). The way risk of bias assessments work is that individuals evaluating a study for internal validity answer a series of signaling questions about the presence or absence of design features followed by a judgment about the potential for the use of the design feature to lead to a biased estimate in the context of the specific study. A conclusion is then reached about potential for bias based on all evaluated design features in the context of the study. Thus, a risk of bias assessment makes fewer assumptions about the link between study design and design features compared to quality assessment. For instance, a quality assessment for an RCT would include an evaluation as to whether blinding of outcome assessors occurred, whereas a risk of bias assessment would involve an evaluation not only as to whether blinding was used, but also a judgement as to whether a lack of blinding of outcome assessors would be likely to lead to a biased estimate given the context of the study and the outcome measures used. Thus, a RCT that did not include blinding of outcome assessors might be rated as poor on a quality assessment but might not be a concern in a risk of bias assessment if the outcomes were measured objectively, precluding the likelihood that the estimate would be biased by a knowledge of the intervention group when classifying the outcomes. Because of the necessity of making a judgement about the potential that bias is associated with design features in the context of a specific topic area, this approach requires the highest level of knowledge of study design and bias. The risk of bias approach also generally is conducted at the outcome level, rather than at the study level. For instance, an unblinded RCT of interventions to treat lameness might be considered to have a high risk of bias if the outcome was assessed by owners (a subjective outcome) but not if the outcome was assessed by force plate measurement (an objective outcome). For a level of evidence assessment, the assessment of internal validity would be high quality because the trial was an RCT. For quality assessment, the study may be considered poor quality because it was unblinded, but the overall judgement would be dependent on a number of other study design flaws identified. Finally, in a risk of bias assessment, the study would likely be low risk of bias for the objective outcome and high risk of bias for the subjective outcome if blinding was not used.

Some components of a risk of bias assessment are the same as those included in a quality assessment approach (e.g., an assessment of randomization, allocation concealment, and blinding could be included in both). However, the way the assessment is done differs, with quality assessments generally involving present/absent judgements as opposed to assessments as to whether the risk of bias is likely or not. Hartling et al. (14) applied two instruments using a quality assessment approach and one instrument using a risk of bias approach to a sample of 163 trials and found that there was low correlation between quality assessment and risk of bias approaches when comparing the assessment of internal validity.

Although the critical elements for risk of bias are well described for RCTs in human healthcare and to a large extent in veterinary RCTs, these elements are not as well described for non-randomized trials and observational studies where allocation to groups is not under the control of the investigator. There are some risk of bias tools available for assessing risk of bias in non-randomized studies, such as ROBINS-I (36). However, ROBINS-I has been criticized for being challenging to use and for having low reliability, particularly amongst less experienced raters (37, 38). A review and critique of approaches to risk of bias assessment for observational studies is available (39). It is anticipated that risk of bias tools for observational study designs, including studies related to questions of prognosis and causation, will continue to evolve as new instruments are developed and validated.

## Historical Contexts and Comparisons of Internal Validity Assessment Approaches

Currently, the available approaches to assessing internal validity tend to be used for different applications. Levels of evidence have previously been used for creating evidence-based recommendations or clinical practices guidelines (30, 40, 41), where it is anticipated that multiple study designs may have been used to address the clinical question(s) of interest. Both quality assessment and risk of bias assessment approaches have been used as a component of systematic reviews with meta-analysis or network meta-analysis, as the intended product of these reviews is to summarize a single parameter (such as incidence or prevalence) or a summary effect size (such as a risk ratio, odds ratio, or hazard ratio) where it is desired that the estimate is unbiased. Often, that estimate is derived from studies with the same study design or a narrow range of study designs from high levels in the evidence hierarchy for the research question type. Therefore, the focus is on a specific parameter estimate based on multiple studies, rather than a descriptive summary of the evidentiary strength of those studies.

However, the different approaches are not necessarily mutually exclusive, but are nested within each other based on assumptions, and the methodology and use of the different approaches has evolved over time. As previously described, a criticism of the use of levels of evidence is that the potential for bias is based on the study design that was employed, rather than the methodological rigor of a specific study (42). For this reason, many frameworks for levels of evidence included wording such as “appropriately designed” (24) or “well designed” (41)for the study designs, although the criteria for determining whether a study was designed and executed with rigor generally is not described. A lack of transparency for the criteria for evaluating internal validity of studies within an evidence level is problematic for individuals wishing to use the results. An example of the evolution toward more transparent considerations of internal validity of individual studies within a levels of evidence framework is seen in the progression of the Australian National Health and Medical Research Council (NHMRC) system for evaluating evidence in the development of clinical practice guidelines. The designation of levels of evidence in this framework originally was based on levels of evidence, with descriptors such as “properly-designed” or “well-designed” included for each type of study design (40). A concern with this approach was that the framework was not designed to address the strength of evidence from individual studies within each evidence level (43). Therefore, the framework was modified to include the use of risk of bias evaluations of individual studies within each evidence level. The combined use of levels of evidence and risk of bias assessment of studies within each level of evidence now forms the “evidence base” component of the NHMRC's FORM framework for the development of evidence-based clinical guidelines (44).

Another example of the evolution of approaches to assessing internal validity is from the Cochrane Back review group, who conduct systematic reviews of neck and back pain. The initial methods guidelines, published in 1997, recommended that a quality assessment be performed on each included study, with each item in the quality assessment tool scored based on whether the authors reported their use (45). Updated methods guidelines were published in 2003 (46). The framework for levels of evidence in this guidance was restricted to a consideration of randomized controlled trials and non-randomized controlled clinical trials, as these were considered the study designs that potentially were appropriate to address research questions in this content area. In the updated guidelines, the recommendations for the assessment of internal validity moved to a risk of bias approach, where judgements were made on whether the characteristics of each study were likely to lead to biased study results. In the 2003 methods guidelines, levels of evidence were recommended as an approach to qualitative analysis rather than the use of “vote counting” (summing the number of studies where a positive or negative outcome was reported). The guidelines were again updated in 2009 (47). In this version, the assessment of the internal validity of individual studies explicitly employed a risk of bias approach. It was further recommended that the use of evidence levels as a component of a qualitative synthesis be replaced with a formal rating of the quality of the evidence for each of the included outcomes. It was recommended that review authors use the GRADE approach for this component. The GRADE approach explicitly includes a consideration of the risk of bias across all studies included in the review, as well as an assessment of the consistency of results across studies, the directness of the evidence to the review question, the precision in the effect size estimate, and the potential for publication bias (48).

## Discussion

The examples from the human medical literature illustrate that assessment of internal validity need not be static, and that modifications to our approach to assessing internal validity can strengthen the evidence base for clinical decision making. When developing or using tools which include an evaluation of internal validity, the assessment of internal validity should use the approach with the least assumptions about bias. This implies that the risk of bias approach, where context specific judgements are made related to the potential for bias, is the preferred approach for assessing internal validity. The risk of bias approach is well developed for RCTs. Therefore, when RCTs are included in the evidence available to address a clinical question, a risk of bias assessment approach should be used. When evaluating internal validity as a component of a SR-MA, the Cochrane ROB2.0 tool (16) could be used for this purpose. Modifications to this tool have been proposed for evaluating trials in livestock trials (49–51). For critical appraisal instruments for RCTs, where additional components such as feasibility and external validity are a desired component, the questions or items within the instrument that are specific to assessing internal validity still could follow a risk of bias approach by specifically requiring a judgement on the potential for bias. Similarly, the use of questions or items requiring a judgement on the potential for bias also could be used for evaluation of RCTs included in clinical practice guidelines when RCTs are present in the evidence base.

However, there are circumstances where these recommendations may not be appropriate or sufficient, such as for observational studies where risk of bias assessment instruments do not formally exist, or where a variety of study designs have been identified that answer the clinical question (particularly non-intervention type questions). When observational studies are used as evidence, individuals assessing internal validity may wish to evaluate risks of bias for each study ad hoc by considering the specific risks of bias related to selection bias, information bias, and confounding in the context of the topic area. However, this approach requires considerable methodological expertise. Alternatively, a quality assessment approach could be used to evaluate internal validity for observational studies, recognizing that more assumptions related to the potential for bias are involved. As instruments for evaluating the risk of bias for observational studies are developed and validated, these could replace ad hoc or quality assessment approaches.

For situations where the evidence base includes multiple study types, such as clinical practice guidelines, the use of levels of evidence may be useful for framing the potential for bias inherent in the studies identified to address the clinical questions. However, within each evidence level, there still is a need to evaluate the internal validity of each study. The proposed approach for situations where RCTs and observational studies are included in the evidence base was described in the preceding paragraphs. For lower levels of evidence, such as case series, textbooks and narrative reviews, and expert opinion, levels of evidence could be used to emphasize that these types of evidence have high potential for bias based on their design.

## Broader Considerations

It should be noted that although this article has focused on approaches to evaluating internal validity of studies, this is only one component of the assessment of evidence. Critical appraisal, CATs, SR-MA, and clinical practice guidelines explicitly incorporate other aspects of decision-making, including a consideration of the magnitude and precision of an intervention effect or the potential clinical impact, the consistency of the research results across studies, the applicability (external validity and feasibility) of the research results, and the directness of the evidence to a clinical situation (for instance, whether the study populations are similar to those in a practice setting). However, a discussion of these components for decision-making is beyond the scope of the current study. The interested reader is referred to further details on the components used in evaluating evidence for CATs (4), for SR-MA using the GRADE approach (52), for network meta-analysis (53) and for clinical practice guidelines (8, 44).

## Author Contributions

JS drafted the manuscript. All authors contributed equally to the conceptualization of this work. All authors read and approved the final contents.

## Funding

Partial funding support was obtained from the University of Guelph Research Leadership Chair (Sargeant).

## Conflict of Interest

The authors declare that the research was conducted in the absence of any commercial or financial relationships that could be construed as a potential conflict of interest.

## Publisher's Note

All claims expressed in this article are solely those of the authors and do not necessarily represent those of their affiliated organizations, or those of the publisher, the editors and the reviewers. Any product that may be evaluated in this article, or claim that may be made by its manufacturer, is not guaranteed or endorsed by the publisher.
